# Hypoglycaemic Unawareness in a Glycogen Storage Disorder Patient: A Case Report and Review of the Literature

**DOI:** 10.7759/cureus.81805

**Published:** 2025-04-06

**Authors:** Alamin Alkundi, Rabiu Momoh

**Affiliations:** 1 Diabetes and Endocrinology, East Kent Hospitals University NHS Foundation Trust, Canterbury, GBR; 2 Critical Care, Medway Maritime Hospital, Gillingham, GBR

**Keywords:** continuous glucose monitoring (cgm), glucose, gycogen, hypoglycaemia, hypoglycaemic unawareness, metabolic disorder, neuroglycopaenic symptoms, patient education, uncooked cornstarch therapy, von gierke disease

## Abstract

The concept of hypoglycaemic unawareness is often discussed in the literature in relation to diabetics on insulin treatment. Hypoglycaemic unawareness is a state in which patients fail to recognize neuroglycopenic symptoms and signs that could prompt the need to seek measures to reverse or treat low blood sugar readings. This can lead to serious dangers, as immediate action is vital to avert potentially life-threatening consequences. We present a finding of hypoglycaemic unawareness in a 27-year-old female with a long-term diagnosis of Von Gierke disease (a glycogen storage disorder (GSD)) who went on to be fitted with a continuous glucose monitoring (CGM) device to prevent the harmful effects of unrecognised hypoglycaemia. We have reviewed the burden and probable mechanism for the development of hypoglycaemic unawareness in this disorder and in other infrequently reported disorders for the education of health professionals and patients.

## Introduction

Glycogen storage disorders (GSDs) are a collection of inherited metabolic conditions caused by enzyme deficiencies that impact glycogen synthesis or breakdown in the liver, muscles and other tissues. These disorders impair the body’s ability to regulate normal blood glucose levels, frequently resulting in episodes of hypoglycemia. Various types of GSDs are known, including type I (Von Gierke disease), which accounts for about 80% of GSD types, type II (Pompe disease), type III (Cori disease) and other rarer types. Von Gierke disease results from a deficiency in the enzyme glucose-6-phosphatase, which is essential for the final steps of gluconeogenesis and glycogenolysis. Patients with GSDs are unable to generate glucose in stressful conditions or fasting states despite having a good store of glycogen and fat in liver and muscle stores. Individuals with Von Gierke disease commonly experience severe hypoglycemia, an enlarged liver (hepatomegaly) and lactic acidosis [1].

The occurrence of hypoglycaemic unawareness is starting to receive attention in the medical literature. It is more often described in relation to diabetics on treatment. Alkundi A et al., in a 2024 publication ,described the occurrence of hypoglycaemic unawareness in a patient with insulinoma. We have gone on to recognize another incidence of hypoglycaemic unawareness in a lady in her late twenties with a GSD [2]. The risk of severe hypoglycaemic brain injury in patients with GSD who develop hypoglycaemic unawareness should then be borne in mind to prevent harmful consequences [3].

## Case presentation

We present the case of a female patient in her late twenties who presented to a general district hospital for recurrent acute vomiting episodes over the course of a 24-hour period. She reported no associated fever, had no respiratory symptoms and reported no urinary symptoms. She was diagnosed with an autosomal recessive inherited glycogen-storage disease type 1a (von Gierke disease) at the age of six months. She was under the care of a metabolic disorder unit at a tertiary centre. Her past hospital records further revealed that she had a learning difficulty, a history of hypoglycaemic coma in childhood and multiple intensive care unit admissions for aspiration pneumonitis due to swallowing difficulty, refractory hypoglycaemia episodes and severe electrolyte derangements. Her regular community prescription included regular dietary uncooked cornstarch therapy and multivitamins, including vitamins D and E and calcium supplement, as well as allopurinol, ferrous fumarate and folic acid. Her mother was her main carer. She lived with her family and had a twice-daily social package of care. She requires a hoist from bed to chair and was fully dependent for activities of daily living. She has regular overnight nasogastric tube feeding tube and regular supplements during the day. She generally maintained normal blood sugar readings with the above feeding regime, and her hypoglycaemic episodes often time with periods of acute illnesses.

Examination on this acute hospital visit revealed an obese patient (weighed 118 kg and body mass index of 58.5 kg/m^2^) who was self-ventilating on room air, pulse oximetry saturation (96%) and respiratory rate 20 cycles per minute. Heart rate was 118 beats per minute, blood pressure was 114/65 mmHg and temperature recorded 36.7 degree Celsius. The capillary refill time was three seconds. Sepsis screen investigations had been done, and intravenous antibiotics had been initiated. Her blood lactate, which had initially been recorded as 17.2 mmol/l (ref: <2 mmol/l) and with intravenous hydration, had improved to 6.2 mmol/l. She also had an intravenous 10% glucose infusing at 167 mls/hr to achieve blood sugar readings between 6 and 10 mmol/l. She was alert and orientated. Her lungs were clear on auscultation, and her abdomen was soft and non-tender. She had an indwelling Portacath line. She required intravenous fluids, intravenous anti-emetics, antibiotics and electrolytes optimization on this admission. After stabilization, she was transferred to a medical ward, and the intravenous dextrose support was weaned off. She was noted with no hypoglycaemic symptoms (even when her blood sugar dropped as low as 1 mmol/l) when her blood glucose readings were monitored on the ward while on her usual feeding protocol. 

**Figure 1:**
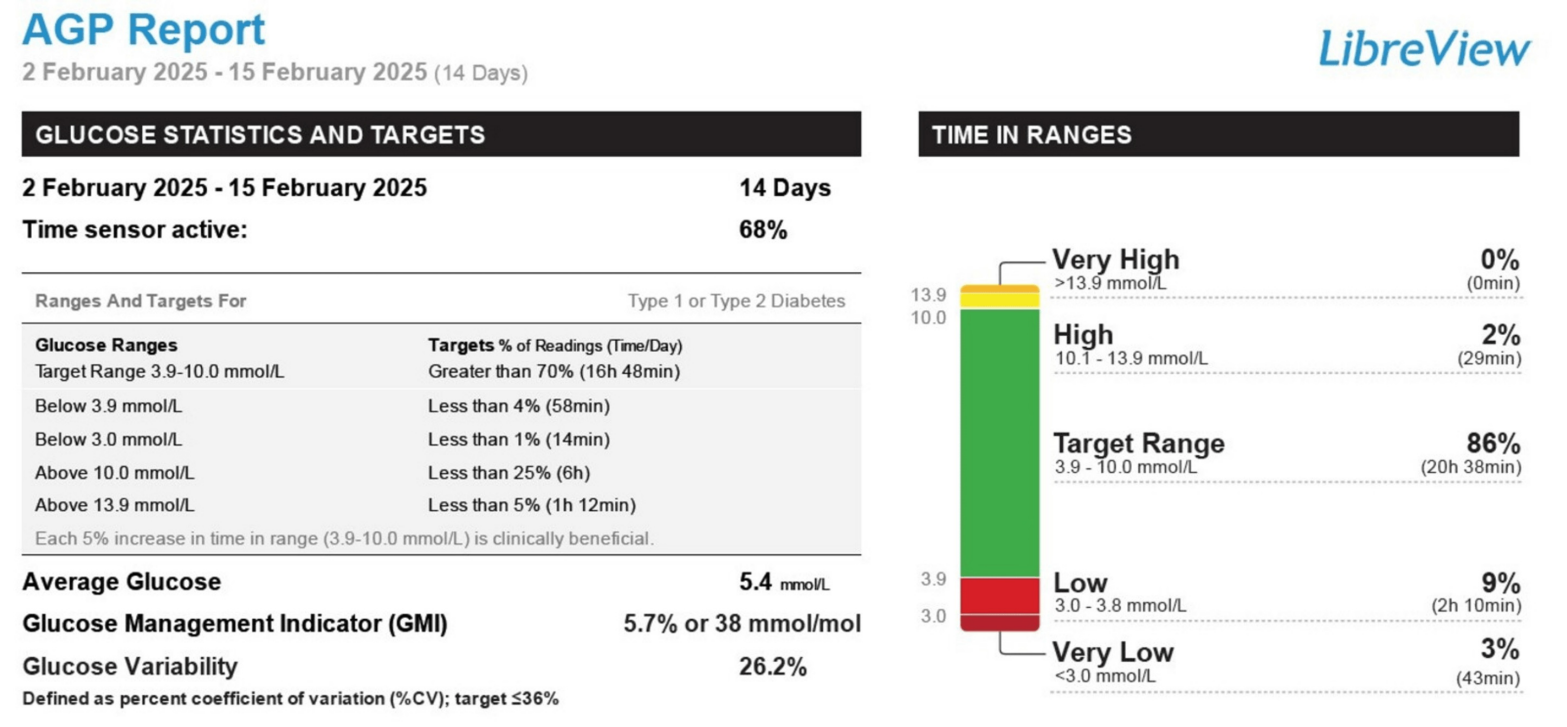
Continuous glucose monitoring report for a two-week period revealing periods spent in hypoglycaemia

**Figure 2:**
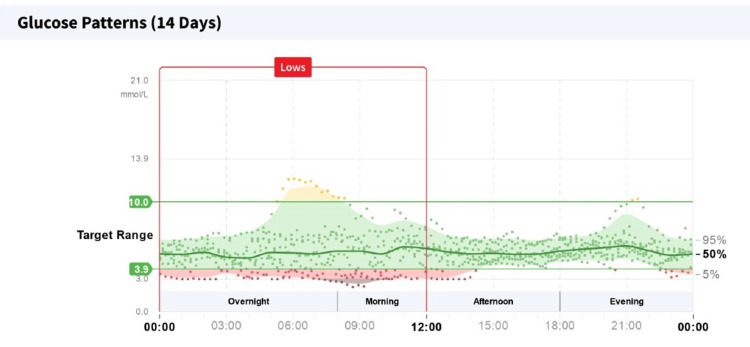
Continuous glucose monitoring report revealing the pattern of hypoglycaemias to be most noted on overnight and morning periods

She was optimized on this hospital visit, and she was initiated on the use of a continuous glucose monitoring (CGM) device (with an alarm feature) for hypoglycaemia monitoring. A two-week report from the CGM device revealed a 12% of the time on monitoring was spent in hypoglycaemia (blood glucose <4 mmol/l), which quantified to two hours and 53 minutes. Of this time, 43 minutes were spent in blood glucose values <3 mmol/l (Figure 1). A significant amount of her hypoglycaemic episodes were recorded overnight and morning hours (Figure 2). A dietetic review was undertaken, and continuous overnight feeding regime via the patient's in-situ nasogastric tube was initiated along with her regular daytime bolus feeding regime.

The patient’s further care was to be continued with input from her community general practitioner and allied community health support services (including physiotherapists, occupational therapists and community dietitians). She was planned for follow-up clinic visits at her regional district hospital under an endocrinology team and with a metabolic disorder team at a tertiary centre.

## Discussion

Hypoglycaemic unawareness occurs when the body's typical physiological reactions to low blood sugar levels are compromised. Normally, a decrease in blood glucose activates the release of counter-regulatory hormones like glucagon and adrenaline, which generate symptoms that alert the person to their condition. However, in cases of hypoglycaemic unawareness, these symptoms can be muted or completely absent [4]. In GSDs, repeated occurrences of hypoglycaemia can lead to a desensitization of the autonomic nervous system, which diminishes the body's ability to indicate low blood sugar levels. An extended duration of a GSD can result in adaptive changes within the body that weaken hypoglycaemic responses [5].

Hypoglycaemic unawareness greatly heightens the risk of severe hypoglycaemia, which may result in seizures, loss of consciousness and even death if not managed quickly. It also impacts the patient’s quality of life, making daily activities, such as driving or exercising, potentially dangerous. The epidemiology of hypoglycaemic unawareness in GSD is yet to be well-described in the literature. Another condition that is receiving attention in terms of its ability to cause hypoglycaemic unawareness is insulinoma. In our index case report, our patient had a known genetic diagnosis of Von Gierke syndrome (a GSD), and she was noted to experience hypoglycaemic unawareness on an in-hospital admission.

Reports of our patient's assessments in childhood that led to this diagnosis are not readily available at the time of this publication. Children are often diagnosed with this disorder as infants. Physical assessments may reveal the presence of hepatomegaly and weak muscles. Blood tests assessing for lactic acidosis, hypoglycaemic readings, hypertriglyceridemia and hyperuricaemia should be assessed. Kidney and liver ultrasound assessments should be done. Genetic study and liver biopsy may be undertaken. Mutation analysis via full gene sequencing of G6PC (GSD Ia) and SLC37A4 (GSD Ib) genes is preferred for confirming the diagnosis of Von Gierke disease. Certain mutations could be missed by this test, and in those cases, quantitative polymerase chain reaction (PCR), long-range PCR, multiplex ligation-dependent probe amplification, targeted array or comparative genomic hybridization analysis could be used. Where liver biopsy is available, assessment of the glucose-6-phosphatase enzyme activity could be done to confirm the diagnosis of Von Gierke disease [6].

A broader overview of hypoglycaemic unawareness in the literature pertains to individuals with diabetes, with type I diabetics being more affected than type II diabetics. Those who are older, have a long history of diabetes or are undergoing intensive diabetes management are also considered to be at higher risk of experiencing hypoglycaemic unawareness [7].

In a study by Christesen HB et al. (2008), a common finding of hypoglycaemic unawareness was noted among four family members with a rare condition known as non-insulinoma persistent hyperinsulinaemic hypoglycaemia (NI-PHH), which is related to a mutation that activates glucokinase [8].

A publication from 1993 by Mitrakou A et al. revealed that insulinoma patients generally exhibited lower levels of counter-regulatory hormones (plasma catecholamines, glucagon, growth hormone and cortisol) during stepped hypoglycaemic-clamp studies when compared to 14 normal individuals matched for age, weight and sex. They observed a normalization of counter-regulatory hormone levels about six months following curative surgeries. In addition, it was noted that untreated insulinoma patients showed a reduction in both autonomic and neuroglycopenic symptoms, as well as a decreased decline in cognitive function during episodes of hypoglycaemia [9]. In a comparative study on type 1 diabetics (divided into two groups of hypoglycaemia aware and unaware patients) done by Sejling et al., they noted that there was no electroencephalogram (EEG) alterations and no change in cognitive performance during hypoglycemia in the two groups during a single hypoglycaemia episode induced by insulin [10].

Total prevention of hypoglycaemia is a crucial strategy to avoid hypoglycaemic unawareness, although achieving this can often be difficult. Monitoring blood glucose levels, establishing personalized goals and providing education are some of the methods available to reach these objectives. Reducing the effects of this condition on GSD patients should be a priority through increased awareness among managing clinicians, enabling early diagnosis of GSD and implementing appropriate supportive care.

CGM devices offer real-time monitoring of blood glucose levels and can alert patients to potential hypoglycaemia before symptoms manifest [11]. Eating frequent small meals: Regular consumption of small, carbohydrate-rich meals aids in maintaining stable blood glucose levels, thereby lowering the likelihood of hypoglycaemia. Collaborating with a dietitian to create a customized meal plan that addresses the specific needs of GSD patients can help avert hypoglycaemic incidents [12].

In certain situations, it may be necessary to modify medications that influence blood glucose levels to lessen the risk of hypoglycaemia. It is vital to educate patients and their families about the signs and dangers of hypoglycaemia and hypoglycaemic unawareness and the significance of consistent monitoring and timely intervention.

Individuals with hypoglycaemic unawareness should always have a source of fast-acting glucose on hand, such as glucose tablets or gel, and wear a medical alert bracelet. They should also possess a glucagon emergency kit, and their family members and close contacts should be trained in how to use it.

## Conclusions

Hypoglycaemic unawareness in individuals with GSDs poses a distinct and difficult clinical situation. By grasping the fundamental mechanisms, recognizing risk factors and applying thorough management strategies, healthcare professionals can reduce risks and enhance the quality of life for these patients. We have gone on to add to the body of literature regarding this burgeoning clinical problem as it affects GSD. More research is suggested to study the increasing problem of hypoglycaemic unawareness complicating endocrinological or metabolic disorders.
